# P-1773. Impact of a Pharmacist-driven Antipseudomonal Antibiotic Time-out

**DOI:** 10.1093/ofid/ofae631.1936

**Published:** 2025-01-29

**Authors:** Carina Diaz, Alice Margulis Landayan, Lorenzo Porras, Sonia Samsundar, Jorge Murillo, Timothy Gauthier

**Affiliations:** South Miami Hospital, Miami, Florida; Baptist Health South Florida, Miami, Florida; South Miami Hospital, Miami, Florida; South Miami Hospital, Miami, Florida; South Miami Hospital, Baptist Health South Florida, Miami, Florida; Baptist Health South Florida, Miami, Florida

## Abstract

**Background:**

Antipseudomonal antibiotics are key weapons in the fight against antimicrobial resistance but misuse can contribute to numerous adverse effects. It is imperative to identify opportunities to further implement strategies for sustained improvement in antipseudomonal antibiotic utilization. The purpose of this quality improvement project was to evaluate the impact of a pharmacist-driven antipseudomonal antibiotic time-out.

**Figure:**
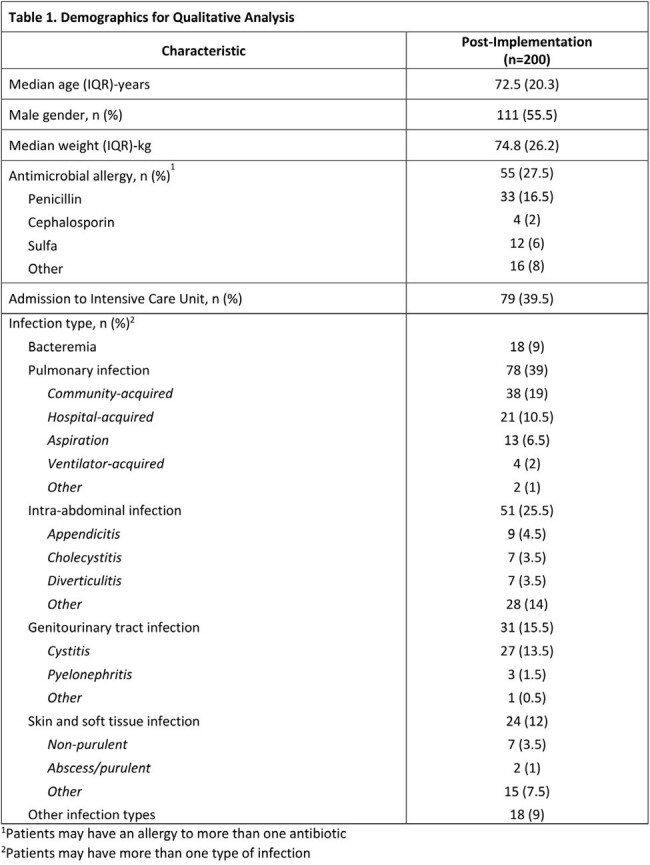

**Methods:**

This was a single-center retrospective evaluation of adults that received piperacillin/tazobactam, cefepime, aztreonam, ciprofloxacin, or levofloxacin while hospitalized. Patients were excluded if pregnant, incarcerated, taking chronic suppressive or prophylactic antimicrobial therapy, receiving hospice care, or on comfort measures only. The primary outcome was the proportion of target antipseudomonal versus all other systemic antibiotic days of therapy adjusted to days present in evaluated units January through February 2023 (pre-implementation) versus January through February 2024 (post-implementation). Qualitative secondary outcomes evaluated for the post-implementation group included pharmacist antibiotic intervention detail, intervention acceptance rate, antipseudomonal agent duration of therapy, incidence of *Clostridioides difficile* infection, and length of hospital stay. The project was Institutional Review Board approved.

**Figure:**
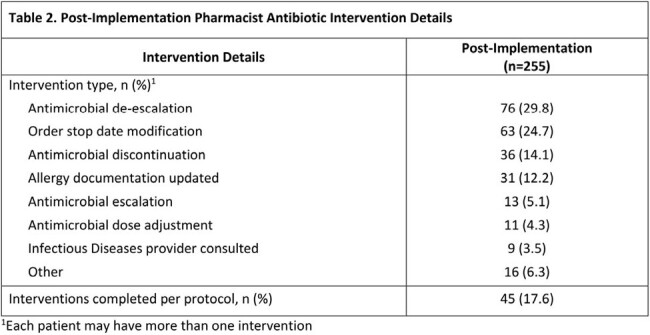

**Results:**

The proportion of target antipseudomonal versus all other systemic antibiotic days of therapy adjusted to days present was 234/898 (26%) for the pre-implementation group versus 210/1321 (16%) for the post-implementation group (p < 0.001). Demographics are listed in table 1. In the post-period 537 patients were reviewed, with 200 patients intervened on and a total of 255 interventions performed with a 99% acceptance rate. The most common types of interventions were antimicrobial de-escalation, order stop date modification, and antimicrobial discontinuation. Pharmacist intervention details are shown in table 2. Other secondary outcomes are displayed in table 3.

**Figure:**
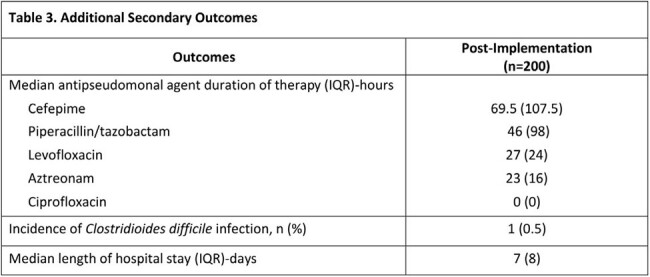

**Conclusion:**

Implementation of a pharmacist-driven antibiotic timeout can drive meaningful reductions in antipseudomonal antibiotic use with high provider acceptance rates without unfavorable clinical outcomes.

**Disclosures:**

**Jorge Murillo, MD**, Paratek Pharma: Advisor/Consultant **Timothy Gauthier, PharmD, BCPS, BCIDP**, AbbVie Pharma: Advisor/Consultant|Antimicrobial Therapy, Inc: Advisor/Consultant|Ferring Pharma: Advisor/Consultant|Firstline Mobile Health: Advisor/Consultant|Gilead Pharma: Advisor/Consultant|GoodRx: Advisor/Consultant|GSK Pharma: Advisor/Consultant|Melinta Pharma: Advisor/Consultant|Pattern Biosciences: Advisor/Consultant|Pfizer Pharma: Advisor/Consultant|ProCE: Honoraria|WWW.LearnAntibiotics.com: Ownership Interest

